# Impact of cerebral protection on observed versus predicted in-hospital stroke in a high stroke risk TAVR cohort

**DOI:** 10.1186/s12872-024-04097-2

**Published:** 2024-08-13

**Authors:** Erez Marcusohn, Ragavie Manoragavan, Stephen Fremes, Christopher Tarola, Janarthanan Sathananthan, Israel M. Barabash, Ady Orbach, Ayaaz K. Sachedina, Sam Radhakrishnan, Harindra C. Wijeysundera

**Affiliations:** 1 Schulich Heart Program, Sunnybrook Health Sciences Centre, University of Toronto, Toronto, Canada; 2 Temerty Faculty of Medicine, University of Toronto, Toronto, Canada; 3 Division of Cardiac Surgery, Sunnybrook Health Sciences Centre, University of Toronto, Toronto, Canada; 4 Centre for Cardiovascular Innovation, St. Paul’s and Vancouver General Hospital, Vancouver, Canada; 5 Cardiovascular Translational Laboratory, Providence Research and Centre for Heart Lung Innovation, Vancouver, Canada; 6 St. Paul’s Hospital, Centre for Heart Valve Innovation, University of British Columbia, Vancouver, BC Canada; 7 Interventional Cardiology Unit, Leviev Heart and Vascular Center, Chaim Sheba Medical Center, Ramat Gan, Israel; 8 Sackler School of Medicine, Tel-Aviv University, Tel Aviv, Israel; 9 Cardiology Department, Edith Wolfson Medical Center, Holon, Israel; 10 Foothills Medical Centre, Libin Cardiovascular Institute, University of Calgary, Calgary, AB Canada; 11 ICES, Toronto, Canada; 12 Institute of Health Policy, Management and Evaluation, University of Toronto, Toronto, Canada

**Keywords:** Cerebral Embolic Protection, Stroke, Aortic stenosis, Transcatheter aortic valve replacement

## Abstract

**Background:**

Despite impressive improvements in the safety profile of Transcatheter aortic valve replacement (TAVR), the risk for peri-procedural stroke after TAVR has not declined substantially. In an effort to reduce periprocedural stroke, cerebral embolic protection (CEP) devices have been utilized but have yet to demonstrate benefit in all-comers. There is a paucity of data supporting the utilization of CEP in TAVR patients with an anticipated high risk for peri-procedural stroke.

**Methods:**

The Transcatheter Aortic Valve Replacement In-Hospital Stroke (TASK) score is a clinical risk tool for predicting the in-hospital stroke risk of patients undergoing transfemoral TAVR. This score was used to identify high-risk patients and calculate the expected in-hospital stroke risk. This was a single-centre cohort study in all consecutive TAVR patients who had placement of CEP. The observed versus expected ratio for peri-procedural stroke was calculated. To obtain 95% credible intervals, we used 1000 bootstrapped samples of the original cohort sample size without replacement and recalculated the TASK predicted scores.

**Results:**

The study included 103 patients. The median age was 83 (IQR 78,89). 63 were male (61.1%) and 45 (43.69%) had a history of previous Stroke or TIA. Two patients had an in-hospital stroke after TAVR (1.94%). The expected risk of in-hospital stroke based on the TASK score was 3.39% (95% CI 3.07–3.73). The observed versus expected ratio was 0.57 (95% CI 0.52–0.64).

**Conclusion:**

In this single-center study, we found that in patients undergoing TAVR with high stroke risk, CEP reduced the in-hospital stroke risk by 43% when compared with the risk-score predicted rate.

**Clinical trial number:**

N/A.

## Introduction

Since the first description of percutaneous Transcatheter aortic valve replacement (TAVR) in 2002, [1] the rapid dissemination of this procedure led to a paradigm shift in therapeutic options for patients with severe aortic stenosis (AS) [2]. It is now the treatment of choice for elderly patients with severe AS, recommended by guidelines [3] for intermediate and high-risk and supported by the Evolut low-risk trial [4] and PARTNER 3 [5] trials for low-risk patients. Correspondingly, TAVR utilization has surpassed surgical aortic valve replacement (SAVR) in many jurisdictions [6, 7]. In particular, there has been an increase in the use of TAVR in patients under 65 years of age [8]. Despite impressive improvements in its safety profile [2] and cost, [9, 10] the risk for peri-procedural stroke after TAVR has not declined substantially since its original clinical trials and remains one of the most feared complications [2, 11, 12].

The average in-hospital stroke rate in patients undergoing TAVR is 1.4–2.1%, with peak incidence on day 2 after the procedure [13–15]. The underlying mechanisms of stroke include the transit of a bulky prosthesis and delivery catheter across an atherosclerotic aortic arch, valvuloplasty of a calcified aortic valve, hypoperfusion causing watershed brain injuries, and human errors related to catheter preparation. The disrupted plaque, thrombi, calcium, or even device material embolize, [16, 17] causing diffusion impairment as shown on brain magnetic resonance imaging (MRI) in between 40 and 84% of procedures [18–20].

As such, efforts to reduce peri-procedural stroke have focused on cerebral embolic protection (CEP) devices. The most used CEP device is the Sentinel Cerebral Embolic Protection Device (Boston Scientific, Marlborough, MA), which is made up of two 140 μm filters positioned in the proximal brachiocephalic artery and proximal left common carotid to capture the debris released throughout the procedure. The device is inserted through a 6-French sheath via the right radial or brachial artery and removed at the end of the procedure [21]. Data on CEP have shown that it can successfully capture debris, [22] is safe, and reduces the MRI volume of cerebral emboli [23]. However, in all comers, it did not reduce clinical stroke, despite a suggestion of a reduction in disabling stroke; moreover, these studies have not identified a single risk factor that confers benefit for CEP [24]. A potential explanation is that the efficacy of CEP may be related to the underlying risk of peri-procedural stroke as defined by the combination of multiple risk factors [14, 24, 25].

Accordingly, to address this knowledge gap, we conducted a single-center study of all consecutive TAVR patients with a high predicted stroke risk who underwent CEP, in order to compare the observed versus expected ratio of in-hospital stroke. Our hypothesis was that CEP would be effective in this higher-risk population.

## Methods

### Study design

A prospective, single-centre cohort study was completed examining all consecutive trans-femoral TAVR patients undergoing CEP at the Schulich Heart Program at Sunnybrook Health Science Centre (SHSC) in Toronto, Ontario, Canada. This is a high-volume tertiary referral hospital that has conducted TAVR since 2009 and performs > 400 TAVR cases per year. In 2020, the Sentinel device was introduced into Sunnybrook. The clinical team only used the device in patients deemed high risk for stroke based on the Transcatheter Aortic Valve Replacement In-Hospital Stroke (TASK) Score TASK score [25].

The study was approved by the Sunnybrook institutional review board and patient consent was waived in light of the fact that CEP was indicated as per current clinical practice by the TAVI team.

### Cohort

All patients who underwent transfemoral TAVR for severe aortic stenosis at SHSC with the Sentinel device from July 2020 to February 2023 were included if the Sentinel device was positioned across at least one major head vessel. Patients were excluded if Sentinel placement was aborted due to technical difficulties, non-transfemoral cases, or procedure indication was Valve in Valve TAVR for degenerative bioprosthetic aortic regurgitation.

### The transcatheter aortic valve replacement in-hospital stroke (TASK) score

The TASK score is a clinical risk tool for predicting the in-hospital stroke risk of patients undergoing transfemoral TAVR [25]. The score was calculated based on a logistic regression model and included peripheral vascular disease (PVD) (OR 1.81 CI 1.18–2.68), use of a non-balloon expandable valve (OR 2.134 CI 1.31–3.5), previous stroke (OR 1.92 CI 1.01–3.19) and chronic kidney disease (CKD) (OR 2.26 CI 1.37–4.03). Each parameter was assigned one point with 4 groups of possible risk; very-low, low, intermediate, and high with 0.7%, 0.8%, 2.1%, and 3.8% risk for stroke respectively. The TASK score was derived using 8779 patients from 12 centers worldwide, with internal validation using bootstrapping. It has intermediate discrimination with a C-statistic of 0.641 and excellent calibration based on the Hosmer-Lemeshow goodness of fit statistic. In this study, we used the logistic model derived from the TASK study to determine the expected risk of in-hospital stroke [25].

### Outcomes

The primary outcome was in-hospital stroke based on evaluation by a neurologist and confirmed according to imaging by CT and/or MRI.

### Variables

Baseline characteristics include age, gender, left ventricular ejection fraction (LVEF), urgency of the procedure, and risk factors such as diabetes, hypertension, dyslipidemia, previous stroke, atrial fibrillation, and CKD (defined as a Glomerular filtration rate of < 60 mL/min/1.73 m2 based on the MDRD equation in the TASK study). Peripheral vascular disease was defined in the TASK study as any documented atherosclerotic disease in the carotid arteries, renal arteries, or any peripheral arteries. That was confirmed with the pre-TAVR CT results. Procedural information included the type of valve, size, and use of pre- and post-balloon valvuloplasty.

### Statistical analysis

The patient’s baseline characteristics were summarized using descriptive statistics. Continuous variables were described using median and interquartile range (IQR). Categorical variables were described using percentages. The expected in-hospital stroke for each patient was calculated according to the TASK score. The expected value for the observed versus expected ratio was calculated as the mean observed risk of stroke divided by the mean expected risk from the TASK score. To obtain 95% credible intervals, we used 1000 bootstrapped samples of the original cohort sample size without replacement and recalculated the TASK predicted scores. Excel version 16.72 was used for the statistical analysis.

## Results

During the study period, 111 patients who underwent transfemoral TAVR were identified as high risk for stroke based on the TASK score and therefore were treated with the Sentinel device. In two patients (1.8%), the Sentinel procedure was aborted due to technical difficulties; in one patient related to difficulty maneuvering through the radial artery and eventual radial perforation, and in the other due to failure to deploy the baskets. Neither had any evidence of a stroke after the procedure. Five patients underwent TAVR valve-in-valve for aortic regurgitation and one patient died at the beginning of the procedure before the TAVR implantation due to LV perforation. After excluding these patients, 103 patients were included in the final analysis.

The median age was 83 (IQR 78,89). 63 patients were male (61.1%) and nine (8.7%) of the procedures were performed urgently in hospitalized patients. Procedural data and risk factors are outlined in Table 1. The median LVEF was 60%, with 18% of patients having an LVEF under 50%. In 13 patients (12.6%), the operator was not able to position the Sentinel device into the left carotid and therefore only positioned the proximal basket.

**Table 1:** Clinical and laboratory characteristics of the study cohort

	Sentinel patients *N* − 103
**Age**,** years**,** median (IQR)**	83 (78,89)
**Male gender**,** n (%)**	63 (61.17%)
**Comorbidities**	
**Diabetes mellitus**,** n (%)**	20 (19.42%)
**Hypertension**,** n (%)**	80 (77.67%)
**Dyslipidemia**,** n (%)**	84 (81.55%)
**Atrial Fibrillation**,** n (%)**	36 (34.95%)
**Ejection Fraction**,** median (IQR)**	60 (52,65)
**TASK variables**	
**Non-balloon expandable valve**,** n (%)**	99 (96.12%)
**GFR < 60 ml/min/1.73m**^**2**^, **n (%)**	11 (10.68%)
**Previous stroke**,** n (%)**	45 (43.69%)
**Peripheral vascular disease**,** n (%)**	103 (100%)
**TAVI procedure**	
**Urgent procedure**,** n (%)**	9 (8.74%)
**Valve type**	
**Evolut**,** n (%)**	90 (87.38%)
**Sapien**,** n (%)**	4 (3.88%)
**Accurate neo**,** n (%)**	9 (8.74%)
**Pre dilatation**,** n (%)**	81(80%)
**Post dilatation**,** n (%)**	25 (24%)
**Outcomes**	
**In hospital stroke**,** n (%)**	2 (1.95%)
**In hospital mortality**,** n (%)**	2 (1.95%)

IQR- interquartile range; GFR – Glomerular filtration rate

### Observed outcomes

Two patients had an in-hospital stroke after TAVR (1.94%). One underwent an MRI with evidence of bilateral multi-territory acute infarcts in which the frontal corona radiata were correlated with the clinical symptoms. The second had a left frontotemporal stroke proven by MRI after unremarkable CT. Of the 13 patients who only had the proximal basket deployed, none had a peri-procedural stroke. There was no evidence of transient ischemic attacks in any of the patients.

Two patients died during hospitalization; one was from sepsis related to ventilator-associated pneumonia, likely from aspiration during emergent resuscitation during the procedure. The other patient died a day after the procedure from probable uncontrolled bleeding, the source of which was not identified.

### Expected stroke

The expected risk of in-hospital stroke based on the TASK score was 3.39% (95% CI 3.07–3.73). Therefore, the observed versus expected ratio was 0.57 (95% CI 0.52–0.64).

## Discussion

In this cohort, we found that CEP reduced in-hospital stroke risk by 43% when used in patients with a high predicted risk of in-hospital stroke. The CEP was positioned with no evidence of complications allowing protection of both carotid arteries in the majority of patients (87.4%).

CEP has been studied in multiple registries and clinical trials. These have shown no benefit for routine use in all comers undergoing TAVR [24, 26, 27]. The largest was the registry analysis of over 123,186 patients, with CEP use in about 15% of patients; the adjusted relative risk with CEP of 0.90 (95% CI, 0.68–1.13) [26]. The recently published PROTECTED TAVR randomized trial failed to show benefit in all stroke events but did show a potential signal for reduction of disabling stroke [24]. Both trials looked at specific subgroups, all of which had similar neutral results as did the main study. Potential explanations might be the small sample size of these subgroups and that they were restricted to a single risk factor.

Our study adds to this literature by specifically focusing on a high-risk cohort, based on the presence of one or more variables from the TASK score. Indeed, our predicted risk was 3.39%, which is substantially higher than the stroke risk seen in most contemporary TAVR trials and closer to the stroke risk in older trials focusing on intermediate -high risk patients [11, 12]. In this cohort, we found that our observed risk was substantially less. We believe that the most likely reason for this, is that the use of CEP mitigated the risk. We hypothesize that we observed this reduction while other studies have not because our overall study had greater power, in part because of the additive nature of the risk factors for stroke. Thus, our predicted baseline stroke risk was much higher compared to the control arms of most trials. There are potentially alternative explanations that merit consideration. One, is that ours is a single centre small cohort study, and our low stroke risk is by chance alone. Second, the patients in our cohort were discharge typically at 24 h after the procedure; therefore, minor strokes and transient ischemic attacks that occurred later at home may have been missed. We cannot discount these possibilities.

The novelty and advantage of the approach we took with our study is that it provides a statistically rigorous evaluation in a clinical situation in which traditional randomized trials will be difficult to conduct. CEP is in everyday use worldwide [2]; indeed, the TVT registry shows that 15% of TAVR cases in the United States had a CEP [2, 26]. As such, from a pragmatic standpoint, future traditional RCTs, in particular those in high-risk populations for stroke, may have major difficulties in recruitment, as the clinical community, from its practice, does not seem to believe there is equipoise. Instead, more creative study designs, such as ours, may be more feasible and therefore should be pursued to provide more evidence in this area.

The relative paucity of evidence for CEP in TAVR patients at high risk for stroke translates to an everyday struggle for most TAVR operators. There is a complex interplay of clinical regret for a potentially avoidable adverse event, in tension with the wish to avoid unnecessary procedures. It is well established that operators are influenced by their patients’ complications and how these could have been prevented. This is particularly true for peri-procedural stroke which can be devastating. This affects operators’ decision-making and patients’ outcomes with a tangible impact on health system costs [28, 29]. The absence of definite evidence, and the low likelihood of future evidence in high-risk subgroups, places the onus on clinicians to determine the best action given the uncertainty. In this case, it is balancing the opportunity cost of additional procedural expense, versus the potential to avoid a stroke, the efficacy of which with CEP, is uncertain. We hope that this study provides additional evidence for clinicians as they deal with this clinical dilemma.

This study has several limitations that merit discussion. First, the TASK score variable definition for PVD is defined as “any documented atherosclerotic disease in the carotid arteries, renal arteries, or any peripheral arteries” [25]. This broad definition included essentially every patient in the cohort. To assess the uncertainty of these estimates, a bootstrap analysis was utilized. This type of analysis can cause overfitting which might result in an overly optimistic estimate. Regarding valve type, our centre usually uses non-balloon expandable valves which were found to increase the risk of stroke and is one of the TASK score variables. Having said that, current literature shows conflicting results on whether the use of a particular prosthesis type increases the risk of stroke. In a propensity score analysis of 171 pairs, balloon-expandable valves had up to 10 times higher risk [30] in small annuli after 1 and 5 years of follow-up and regardless of specific anatomy in others [31]. However, another large study comparing the two showed opposite results [32]. Risk scores for the TAVR procedure itself were not collected routinely during the study and are not presented in the results section as TAVR is approved for all risk levels in Ontario. Finally, this study was a single center and relatively small cohort – it should therefore be considered hypothesis generating and not conclusive.

In summary, we found that in patients undergoing TAVR with high stroke risk, CEP reduced the in-hospital stroke risk by 43% when compared with the risk-score predicted rate and reinforced the need for further study in this area with more pragmatic trial designs.

## Data Availability

The datasets used and/or analysed during the current study are available from the corresponding author on reasonable request.

## Abbreviations

TAVR: Transcatheter aortic valve replacement
CEP: Cerebral embolic protection
AS: Aortic stenosis
SAVR: Surgical aortic valve replacement
CT: Computerized tomography
MRI: Magnetic resonance imaging
TASK score: The Transcatheter Aortic Valve Replacement In-Hospital Stroke Score
IQR: Interquartile range
TIA: Transient ischemic attack
CKD: Chronic kidney disease
PVD: Peripheral vascular disease
OR: Odds ratio
RCT: Randomized controlled trial
CI: Credible interval
